# Anti-Typhoid Inoculation

**Published:** 1901-11-09

**Authors:** 


### ANTI-TYPHOID INOCULATION.

The discussion on a paper read by Drs. Elliot and Washbourn at the last meeting of the Medical Society elicited various opinions as to the efficacy of inoculation as a preventive of typhoid fever. The authors themselves did not consider that the results pointed to any marked beneficial influence of inoculation, while from the cases which occurred in their staff they drew the conclusion that it was not of any practical value in preventing the disease. Dr. William Cayley, however, thought that the evidence in favour of the immunity conferred by inoculation was sufficiently strong to make it desirable that it should continue to be used. Professor Wright, of course, was in favour of this being done, but he described the precautions which should be taken, as recently detailed in The Hospital (September 28th). Surgeon-General Henry Cayley thought that the evidence proved the value of inoculation for a certain time, possibly from five to six months, and he believed that inoculation made the attacks less severe. Dr. Howard Tooth said that although his figures were small they were favourable to inoculation. He thought that the more severely the person reacted to the inoculation, the less likely was he to be immune. In Bloemfontein officers who had had severe reaction did not seem to be protected; in which he would seem to be in accord with Professor Wright. Dr. H. D. Rolleston believed there were several different strains of the typhoid bacillus, and that an individual infected with one strain was not proof against other strains, a doctrine which evidently cuts at the root of the specificity of enteric fever.

